# Using matrix assisted laser desorption ionisation mass spectrometry (MALDI-MS) profiling in order to predict clinical outcomes of patients with heart failure

**DOI:** 10.1186/s12014-018-9213-1

**Published:** 2018-11-02

**Authors:** Thong Huy Cao, Donald J. L. Jones, Paulene A. Quinn, Daniel Chu Siong Chan, Narayan Hafid, Helen M. Parry, Mohapradeep Mohan, Jatinderpal K. Sandhu, Stefan D. Anker, John G. Cleland, Kenneth Dickstein, Gerasimos Filippatos, Hans L. Hillege, Marco Metra, Piotr Ponikowski, Nilesh J. Samani, Dirk J. Van Veldhuisen, Faiez Zannad, Aeilko H. Zwinderman, Adriaan A. Voors, Chim C. Lang, Leong L. Ng

**Affiliations:** 1 Department of Cardiovascular Sciences, University of Leicester and National Institute for Health Research Leicester Biomedical Research Centre, Glenfield Hospital, Leicester, LE3 9QP UK; 2 Leicester Cancer Research Centre, Leicester Royal Infirmary, University of Leicester, Leicester, UK; 3 Division of Molecular and Clinical Medicine, Ninewells Hospital and Medical School, University of Dundee, Dundee, DD1 9SY UK; 4 Division of Cardiology and Metabolism, Department of Cardiology (CVK), and Berlin-Brandenburg Center for Regenerative Therapies (BCRT), German Centre for Cardiovascular Research (DZHK) partner site Berlin, Charité Universitätsmedizin Berlin, Berlin, Germany; 5 Robertson Centre for Biostatistics, Institute of Health and Wellbeing, University of Glasgow, Glasgow Royal Infirmary, Glasgow, UK; 6 University of Bergen, Stavanger University Hospital, Stavanger, Norway; 7 Department of Cardiology, Heart Failure Unit, Athens University Hospital Attikon, School of Medicine, National and Kapodistrian University of Athens, Athens, Greece; 8 Department of Cardiology, University of Groningen, Groningen, The Netherlands; 9 Department of Medical and Surgical Specialties, Radiological Sciences and Public Health, Institute of Cardiology, University of Brescia, Brescia, Italy; 10 Department of Heart Diseases, Wroclaw Medical University, Wroclaw, Poland; 11 Cardiology Department, Military Hospital, Wroclaw, Poland; 12 Inserm CIC 1433, Université de Lorrain, CHU de Nancy, Nancy, France; 13 National Heart Centre Singapore, Singapore, Singapore

**Keywords:** MALDI-MS, Heart failure, Biomarker, Clinical outcome, Proteomics

## Abstract

**Background:**

Current risk prediction models in heart failure (HF) including clinical characteristics and biomarkers only have moderate predictive value. The aim of this study was to use matrix assisted laser desorption ionisation mass spectrometry (MALDI-MS) profiling to determine if a combination of peptides identified with MALDI-MS will better predict clinical outcomes of patients with HF.

**Methods:**

A cohort of 100 patients with HF were recruited in the biomarker discovery phase (50 patients who died or had a HF hospital admission vs. 50 patients who did not have an event). The peptide extraction from plasma samples was performed using reversed phase C18. Then samples were analysed using MALDI-MS. A multiple peptide biomarker model was discovered that was able to predict clinical outcomes for patients with HF. Finally, this model was validated in an independent cohort with 100 patients with HF.

**Results:**

After normalisation and alignment of all the processed spectra, a total of 11,389 peptides (m/z) were detected using MALDI-MS. A multiple biomarker model was developed from 14 plasma peptides that was able to predict clinical outcomes in HF patients with an area under the receiver operating characteristic curve (AUC) of 1.000 (p = 0.0005). This model was validated in an independent cohort with 100 HF patients that yielded an AUC of 0.817 (p = 0.0005) in the biomarker validation phase. Addition of this model to the BIOSTAT risk prediction model increased the predictive probability for clinical outcomes of HF from an AUC value of 0.643 to an AUC of 0.823 (p = 0.0021). Moreover, using the prediction model of fourteen peptides and the composite model of the multiple biomarker of fourteen peptides with the BIOSTAT risk prediction model achieved a better predictive probability of time-to-event in prediction of clinical events in patients with HF (p = 0.0005).

**Conclusions:**

The results obtained in this study suggest that a cluster of plasma peptides using MALDI-MS can reliably predict clinical outcomes in HF that may help enable precision medicine in HF.

**Electronic supplementary material:**

The online version of this article (10.1186/s12014-018-9213-1) contains supplementary material, which is available to authorized users.

## Background

Biomarkers play a major role in the management of patients with heart failure (HF) with established roles in diagnosis, prognosis, risk stratification and guiding therapy. In addition, biomarkers have been shown to be useful in understanding the pathophysiology of HF, particularly in specific phenotypes. Therefore, finding novel biomarkers might further improve our understanding and management of HF [1].

Matrix assisted laser desorption ionisation mass spectrometry (MALDI-MS) has emerged into an important proteomic technology, which has been used for analysing plasma proteomic spectra [2–9]. MALDI-MS analysis offers a highly sensitive method for discovery of biomarkers directly from complex biological fluids such as plasma.

To the best of our knowledge, there has not been any study using MALDI-MS technology that enables the detection of novel biomarkers predicting clinical outcomes in patients with HF. The main aim of this study was to develop a plasma peptide model that would enable better prediction of clinical outcomes in patients with HF. In this turn, this may help increase our understanding of the pathophysiology of HF.

## Methods

### Patient population

Patients were selected from the BIOSTAT-CHF (A systems BIOlogy Study to TAilored Treatment in Chronic Heart Failure) project which was an investigator-driven multicentre clinical study [10]. The main aim of this project was to identify poor outcomes in HF patients with a standard treatment using a systems biology approach which includes demographics, biomarkers, genetics and proteomics [11, 12]. The BIOSTAT-CHF project was conducted according to the declaration of Helsinki which was approved by national and local ethics committees. All patients provided written informed consent. Participating subjects who met inclusion and exclusion criteria according to the European Society of Cardiology (ESC) guideline were collected [13]. In brief, 2516 patients were more than 18 years old, presented symptoms of HF and had left ventricular ejection fraction (LVEF) ≤ 40% and/or B-type natriuretic peptide (BNP) > 400 pg/mL or N-terminal pro B-type natriuretic peptide (NT-proBNP) > 2000 pg/mL who were recruited into the BIOSTAT-CHF project. At the beginning of the study, blood samples were collected for proteomic analysis. Blood was drawn by venepuncture that were obtained from supine patients after at least 15 min bed rest. Blood was collected in 10 mL EDTA vacutainer tubes, inverted 8 times and put on ice immediately. Plasma was obtained after centrifugation at 1000*g* for 15 min at 4 °C, transferred to small aliquots and stored at − 80 °C until further analysis. Then the patients received a standard therapy for HF which included up-titration with angiotensin converting enzyme inhibitors and beta blockers from 0 to 6 months to optimise the treatment. Clinical events such as death and HF hospitalisation were followed. The plasma sample groups were sex and age matched. In the biomarker discovery phase, one group consisted of 50 patients with HF (25 male and 25 female) who died or were rehospitalised for HF, and they were compared with the group of 50 HF patients who did not have such an event (Table 1). A separate cohort of a hundred HF patient plasma samples from the BIOSTAT-CHF project [10] that was employed for verification in the biomarker validation phase in this study (Table 2).

**Table 1:** Patient characteristics of biomarker discovery HF patient cohort

Characteristics	HF hospitalisation or death (n = 50)	No event (n = 50)	p value
Age (years)	76.64 ± 8.14	76.64 ± 8.14	1.000
Male sex, n (%)	25 (50)	25 (50)	1.000
BMI (kg/m^2^)	30.01 ± 6.17	28.94 ± 6.66	0.471
NYHA class III/IV, n (%)	38 (76)	27 (54)	*0.021*
Systolic blood pressure (mmHg)	126.38 ± 20.63	130.94 ± 21.12	0.247
Diastolic blood pressure (mmHg)	66.92 ± 11.92	69.22 ± 12.24	0.324
Heart rate (bpm)	75.69 ± 19.91	73.94 ± 18.28	0.848
HF hospitalisation/Death	32/18	0/0	
Serum creatinine (µmol/L)	126.88 ± 58.56	107.16 ± 34.27	0.076
eGFR (mL/min^−1^)	45.76 ± 14.23	51.34 ± 11.19	*0.037*
Primary aetiology			
Ischemic heart disease	40 (80)	32 (64)	0.118
Non ischemic heart disease	10 (20)	18 (36)	0.118

Italic values indicate significance of p value (p < 0.05)

*BMI* body mass index, *eGFR* estimated glomerular filtration rate, *NYHA* New York Heart Association

**Table 2:** Patient characteristics of the biomarker validation HF patient cohort

Characteristics	HF hospitalisation or death (n = 58)	No event (n = 42)	p value
Age (years)	69.52 ± 12.15	68.86 ± 11.95	0.696
Male sex, n (%)	29 (50.0)	20 (47.6)	0.814
BMI (kg/m^2^)	27.76 ± 6.20	29.27 ± 5.85	0.125
NYHA class III/IV, n (%)	36 (65.5)	27 (64.3)	0.905
Systolic blood pressure (mmHg)	125.10 ± 25.20	123.52 ± 17.07	0.936
Diastolic blood pressure (mmHg)	72.00 ± 14.41	75.50 ± 11.48	0.054
Heart rate (bpm)	82.53 ± 22.37	83.55 ± 24.52	0.975
BNP (pg/mL)	467.45 ± 433.66	288.49 ± 390.02	*0.004*
Serum creatinine (µmol/L)	123.72 ± 47.06	101.32 ± 46.09	*0.004*
eGFR (mL/min^−1^)	53.74 ± 20.03	67.63 ± 27.72	*0.013*
Primary aetiology			
Ischaemic heart disease	27 (47.4)	15 (36.6)	0.287
Non ischaemic heart disease	30 (52.6)	26 (63.4)	0.287

Italic values indicate significance of p value (p < 0.05)

*BMI* body mass index, *BNP* brain natriuretic peptide, *eGFR* estimated glomerular filtration rate, *NYHA* New York Heart Association

### Sample preparation

#### Peptide extraction

Reversed phase C_18_ (C_18_ extra wide pore solid phase extraction cartridges) was used to capture peptides in plasma samples. C_18_ EWP SPE cartridges were primed with 1 column volume (3 mL) of methanol and then washed with 2 column volumes (6 mL) of 18.2-MΩ-cm deionised water before washing with 2 column volumes of 0.1% formic acid (FA). 100 μL of each plasma sample were mixed with 1 mL of 1% trifluoroacetic acid (TFA) and left on ice for 20 min to allow precipitation. Then, the sample was centrifuged at 14,000*g* for 10 min at 4 °C. 950 µL of the dissolved sample was applied on a C_18_ EWP SPE cartridge. Each cartridge was washed with 2 column volumes of 0.1% formic acid and then 2 column volumes of water. Peptides were eluted by adding 1.2 mL elution solution of 60% acetonitrile (ACN) + 0.1% formic acid (FA) in water and then 1.2 mL of 90% acetonitrile + 0.1% formic acid in water. Finally, the eluates were dried by using a Speed-Vac (Jouan, Thermo Scientific, USA) for 2 h and followed by freeze-drying overnight (Edwards, Modulyo, BPS, UK). The samples were stored at − 80 °C until MALDI-MS analysis.

#### MALDI spot preparation

The dried samples were reconstituted in 0.1% trifluoroacetic acid (TFA). 10 µL of each sample were mixed with 990 µL of α-CHCA matrix solution (5 mg α-cyano-4-hydroxycinnamic acid in 1 mL of 50% acetonitrile + 50% water with 0.1% trifluoroacetic acid). Then, 1 µL of this mixture was spotted in triplicate directly onto a 96 well MALDI target plate (Waters Corporation, Manchester, UK). The target plates were dried at room temperature for 45 min and immediately transferred into the MALDI-MS for analysis.

### Sample analysis

Samples were analysed using a Synapt G2 MALDI mass spectrometer (Waters Corporation, Manchester, UK) tuned to 10,000 mass resolution (full width at half height). The MALDI-MS instrument and mass spectra were automatically acquired in positive mode. Peptides were detected in a mass range of m/z from 700 to 10,000 using instrument settings optimised for plasma analysis with the following acquisition settings: plate speed: 15, laser firing rate: 200, laser energy: 300, mass threshold: 10. Ionisation was performed with a laser operating at a frequency of 1000 Hz. For each MALDI spot, spectra were recorded from vertical spot positions.

### Data analysis

Raw data files were converted to txt files using MassLynx version 4.1 software (Waters Corporation, Manchester, UK) before they were imported into Progenesis MALDI version 1.4 software (Nonlinear Dynamic, UK). Spectra were pre-processed to remove noise and background across all spectra: a noise filter size of 5 was applied and background subtracted using a top hat filter size of 60. All the features in spectra were aligned using a search area of 5 before analysis. The data obtained was exported to Excel for further analysis.

### Statistical analysis

All data for continuous variables are reported as mean ± SD. After testing for normal distribution, values were compared by unpaired Student’s t tests or Mann–Whitney U test, as appropriate. All statistical tests were performed 2-tailed, and a significance level of p value < 0.05 was considered to indicate statistical significance. To evaluate test performance of candidate biomarkers as predictors for outcomes in patients with HF, the area under the receiver operating characteristic curves (AUC) were plotted. The multiple biomarker model were built using a logistic regression with candidate peptides (m/z) which were entered simultaneously in order to improve the predictive probability of outcomes in patients with HF. The SPSS statistics software version 24.0 (Statistical Package for the Social Sciences, Chicago, USA) for Windows was employed for all statistical analyses in this study.

## Results

### Patient characteristics

Patient characteristics of the biomarker discovery HF cohort are described in Table 1. In the biomarker discovery HF patient cohort, the groups were matched in age (average age: 76.6 ± 8.1 years old) and gender between both HF groups. The age and gender distribution of both groups of patients with HF was not statistically different (p = 1.000). Therefore, age and gender bias was completely excluded.

Patient characteristics of the biomarker validation HF patient cohort are displayed in Table 2. Mean age was 69.5 ± 12.2 years in the patients who died or were re-hospitalised and 68.9 ± 12.0 years in the patients who did not have an event (p = 0.696). In the patients with an event, eGFR (mL/min^−1^) was lower (53.74 ± 20.03 vs. 67.63 ± 27.72, p = 0.013 and BNP levels (pg/mL) were higher (467.45 ± 433.66 vs. 288.49 ± 390.02, p = 0.004). All other patient characteristics were not significantly different between the two HF groups.

### Identification of plasma peptide spectra in patients with heart failure

We analysed the plasma peptide profiles of a hundred patients with HF in the biomarker discovery cohort and a hundred patients with HF in the biomarker validation cohort. After normalisation and alignment of all the processed spectra, a total of 11,389 peptides (m/z) were detected using MALDI-MS combined with C_18_ SPE. From the 11,389 peptides, expression of 53 peptides (m/z) were significantly different in both cohorts in HF patients with and without an event at a p value < 0.05 (Table 3).

**Table 3:** List of 53 peptides (m/z) detected in both biomarker discovery and validation HF patient cohorts which were significantly different in expression in the patients with HF who responded to treatment as compared to the HF hospitalisation/death at p value < 0.05

m/z	Fold change	p value
1724.22	0.97	0.034
2279.24	0.94	0.028
2290.24	0.95	0.043
2300.24	0.95	0.029
2410.29	0.95	0.028
2472.34	0.94	0.028
2646.44	1.06	0.019
2691.47	0.93	0.007
2729.47	0.94	0.037
2868.59	1.08	0.018
3113.71	1.07	0.042
5636.08	1.43	0.041
5660.99	1.31	0.049
5855.33	0.82	0.030
5953.32	1.58	0.009
6165.30	1.60	0.036
6279.13	2.26	0.023
6283.58	1.45	0.014
6314.83	1.49	0.031
6446.94	1.24	0.043
6460.55	2.00	0.027
6465.03	2.98	0.004
6515.90	0.38	0.001
6551.62	1.52	0.041
6576.58	1.99	0.004
6576.99	1.55	0.010
6601.97	1.63	0.045
6609.77	1.52	0.047
6722.04	1.61	0.009
6764.13	1.60	0.025
6918.14	1.63	0.028
7061.32	2.99	0.040
7100.13	3.22	0.027
7118.44	1.83	0.037
7121.74	1.69	0.048
7158.59	2.77	0.045
7185.63	2.10	0.028
7213.01	2.17	0.028
7358.59	3.76	0.011
7409.39	0.92	0.002
7463.58	1.74	0.013
7479.14	0.44	0.003
7492.90	0.58	0.027
7526.71	1.60	0.048
7572.41	1.84	0.036
7582.00	5.29	0.016
7600.74	2.25	0.018
7634.93	1.81	0.013
7649.22	3.10	0.033
7889.48	1.66	0.033
7914.92	2.78	0.005
7928.13	0.31	0.006
7929.78	3.25	0.028

### Selection of candidate peptide (m/z) biomarkers for prediction of clinical outcomes in the biomarker discovery phase

**Fig. 1:**
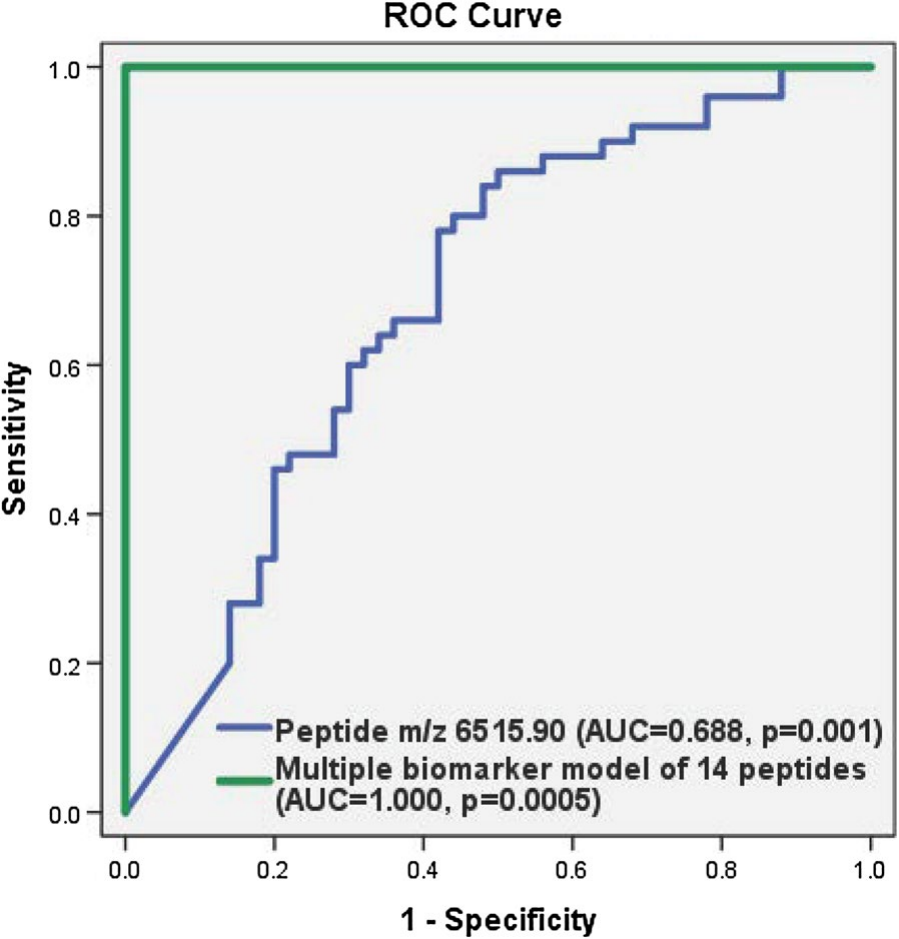
Receiver operating characteristic (ROC) curve of peptide m/z 6515.90 and the multiple biomarker model of fourteen peptides for prediction of clinical outcomes in the biomarker discovery HF patient cohort. The blue curve displays the best AUC with a single biomarker was peptide m/z 6515.90 with AUC of 0.688 (Asymptotic 95% confidence interval [CI], 0.583–0.793, p = 0.001) in discriminating the HF patients who respond to treatment from HF hospitalisation/death. The green curve shows a multiple biomarker model with fourteen peptides (m/z 2646.44, 2729.47, 3113.71, 5636.08, 5855.33, 5953.32, 6314.83, 6465.03, 6515.90, 7061.32, 7358.59, 7492.90, 7582.00 and 7929.78) with an excellent improvement in the performance of predictive probability for clinical outcomes in patients with HF with an AUC of 1.000 (Asymptotic 95% CI, 1.000–1.000, p = 0.0005)

To determine if peptides (m/z) could help to discriminate clinical outcomes between the HF patients with or without an event, receiver operating characteristic (ROC) curves were generated. Additional file 1: Table S1 shows the values of area under the receiver operating characteristic curves (AUC) for 53 peptides (m/z). The best AUC was peptide m/z 6515.90 with AUC of 0.688 at p = 0.001 (Asymptotic 95% confidence interval [CI], 0.583—0.793) that is presented in Fig. 1.

However, no individual peptide (m/z) was an excellent classifier for prediction of clinical outcomes in patients with HF. Therefore, the development of a multiple peptide biomarker approach would be useful to provide more pathophysiological information about patients with HF and able to predict clinical outcomes. We developed a multiple biomarker model with fourteen peptides (m/z 2646.44, 2729.47, 3113.71, 5636.08, 5855.33, 5953.32, 6314.83, 6465.03, 6515.90, 7061.32, 7358.59, 7492.90, 7582.00 and 7929.78) by using a logistic regression in which all these peptides were entered simultaneously (Additional file 1: Table S1 and Additional file 2: Figure S1). The AUC value in the multiple biomarker model of fourteen peptides showed an excellent improvement in the performance of predictive probability for clinical outcomes in patients with HF with an AUC of 1.000 (Asymptotic 95% CI, 1.000–1.000) at p = 0.0005. The prediction capability of this model achieved 100% sensitivity and 100% specificity (Fig. 1). There was a very good separation between the HF patients who responded to treatment and HF hospitalisation or death which is displayed in a scatter 3D plot of fourteen peptide model (Additional file 3: Figure S2).

### Validation of candidate peptide (m/z) biomarkers for prediction of clinical outcomes in the biomarker validation phase

**Fig. 2:**
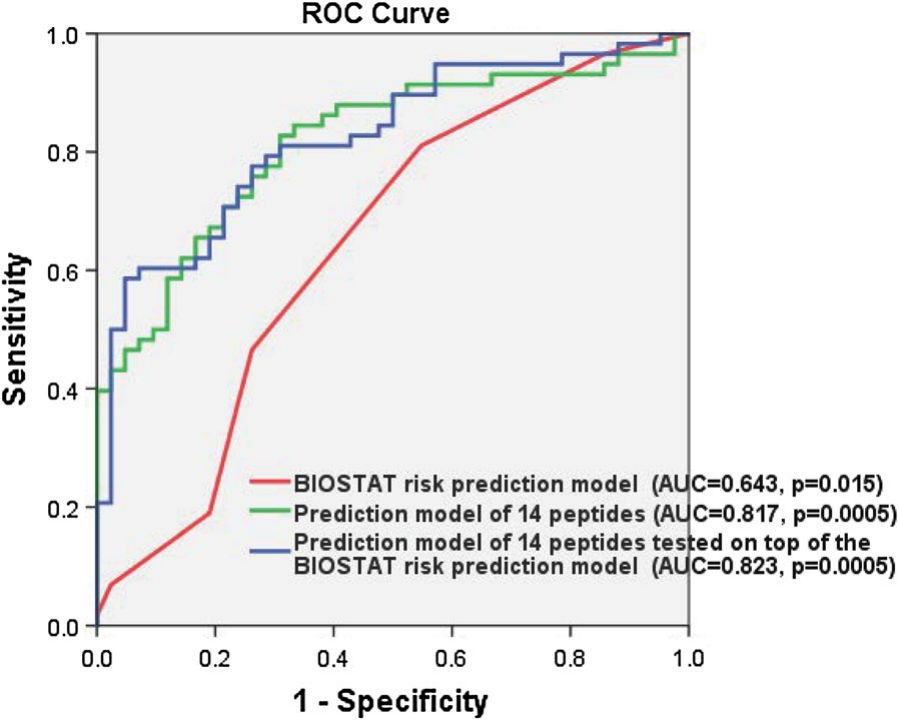
Receiver operating characteristic (ROC) curve of the multiple biomarker model with fourteen peptides for prediction of clinical outcomes of HF in comparison with the BIOSTAT risk prediction model and the added value of the prediction model of fourteen peptides on top of the BIOSTAT risk prediction model. The red curve presents the BIOSTAT risk prediction model with an AUC value of 0.643 (Asymptotic 95% CI 0.530–0.757, p = 0.015) that risk scores were calculated using the online calculator available at: http://www.biostat-chf.eu (including age, HF hospitalisation last year, peripheral oedema, systolic blood pressure, NT-proBNP, haemoglobin, high-density lipoprotein, serum sodium and beta-blocker use at baseline). The green curve describes the multiple biomarker model with the fourteen peptides with an AUC of 0.817 (Asymptotic 95% CI 0.734–0.900, p = 0.0005). The blue curve displays the prediction model of fourteen peptides on top of the BIOSTAT risk prediction model with an AUC of 0.823 (Asymptotic 95% CI 0.743–0.904, p = 0.0005)

To confirm the result achieved in the biomarker discovery phase, the multiple biomarker model with a combination of fourteen peptides discovered from the biomarker discovery HF patient cohort was tested in the biomarker validation HF patient cohort with another hundred patients with HF. The AUC value of this multiple biomarker model with the fourteen peptides yielded an AUC of 0.817 at the p value of 0.0005 (Asymptotic 95% CI 0.734–0.900) that is shown in Fig. 2 and Table 4.

**Table 4:** AUC values of the multiple biomarker model of fourteen peptides for prediction of clinical outcomes in the biomarker validation HF patient cohort in comparison with the BIOSTAT risk prediction model and the added value of the prediction model of fourteen peptides on top of the BIOSTAT risk prediction model

m/z	AUC	Standard error	p value	Asymptotic 95% confidence interval	
				Lower bound	Upper bound
BIOSTAT risk prediction model	0.643	0.058	0.015	0.530	0.757
Prediction model of 14 peptides	0.817	0.042	0.0005	0.734	0.900
Prediction model of 14 peptides tested on top of the BIOSTAT risk prediction model	0.823	0.041	0.0005	0.743	0.904

### The added value of the multiple peptide biomarker model on top of the BIOSTAT risk prediction model

Recently, we developed a risk prediction model for patients with HF from the BIOSTAT-CHF cohort [14] which risk scores can be calculated using the online calculator available at: http://www.biostat-chf.eu (including age, HF hospitalisation last year, peripheral oedema, systolic blood pressure, NT-proBNP, haemoglobin, high-density lipoprotein, serum sodium and beta-blocker use at baseline). Using the BIOSTAT risk prediction model generated an AUC value of 0.643 (Asymptotic 95% CI 0.530–0.757) with p value of 0.015 (Fig. 2 and Table 4). Interestingly, the added value of the prediction model of fourteen peptides on top of the BIOSTAT risk prediction model achieved an AUC of 0.823 (Asymptotic 95% CI 0.743–0.904, p = 0.0005) that is displayed in Fig. 2 and Table 4. The increase in the AUC value of the composite model of the BIOSTAT risk prediction model with the multiple peptide model as compared to the BIOSTAT risk prediction model had a statistically significant p value of 0.0021. In addition, using the prediction model of fourteen peptides and the composite model of the multiple biomarker of fourteen peptides with the BIOSTAT risk prediction model gave a better predictive probability of time-to-event in prediction of clinical events in patients with HF (p = 0.0005, Additional file 4: Figure S3).

## Discussion

There is no single effective parameter to predict clinical outcomes in patients with HF. Therefore, several models have been applied to predict mortality and HF hospitalization in patients with HF. In a meta-analysis, the mean c-statistics of all of these models to predict mortality and/or HF admission was only 0.63 [15]. Recently, we developed a risk prediction model from the BIOSTAT-CHF cohorts, which yielded a c-statistics of 0.71 to predict death or HF admission [14]. Therefore, a method to enable clinicians to better predict clinical outcomes in HF would be important and useful for improving prognostication and in stratifying patients with HF. Using the MALDI-MS technique for proteomic analysis is one of the most promising approaches for the discovery and identification of peptides and proteins in many diseases. Based on this technology, some biomarkers of several diseases have been discovered, particularly in cancer [2–9]. Thus we sought to see if we could devise a strategy to combine MALDI-MS and C_18_ SPE technique and employ statistical tools to establishing a model that could discriminate between HF patients who respond to treatment and HF hospitalisation or death.

In this study, a total of 11,389 peptides (m/z) were detected using MALDI-MS combined with C_18_ SPE in both biomarker discovery and validation HF patient cohort. Moreover, 53 peptides showed a significantly different expression between patients who died or had a HF admission and those who did not have such an event. These results demonstrated that MALDI-MS profiling could be used to discriminate between HF patients with and without clinical events. These peptides correspond to small proteins or fragments of proteins in plasma that might have important roles in the pathogenesis of the HF.

The change in expression of peptides reflects changes in plasma which could potentially be due to pathophysiological processes in HF. Thus, it is unlikely that there would be a single peptide which could be able to identify clinical outcomes in patients with HF. With a single biomarker, peptide m/z 6515.90 gave the best AUC value of 0.688 (p = 0.001) in discriminating the HF patients who respond to treatment from HF patients with death/rehospitalisation (Fig. 1). However, due to the heterogeneity of clinical populations (age, sex, ethnicity and comorbidity) an ideal single biomarker may not exist for each disease [16]. Some reports have demonstrated that a panel of multiple potential biomarkers in a specific model could improve precision and be more robust [17–19]. Therefore, we developed a multiple biomarker model with a cluster of peptides (m/z) that would provide better prediction of clinical outcomes for patients with HF. The performance of this multiple biomarker model was much better as compared to each single peptide biomarker for prediction of clinical outcomes in patients with HF (Fig. 1 and Table 4). The multiple biomarker model with fourteen peptides (m/z 2646.44, 2729.47, 3113.71, 5636.08, 5855.33, 5953.32, 6314.83, 6465.03, 6515.90, 7061.32, 7358.59, 7492.90, 7582.00 and 7929.78) gave an excellent area under the ROC curve of 1.000, p = 0.0005 (Fig. 1). This discrimination value was maintained with an AUC of 0.817 (p = 0.0005) in the biomarker validation HF patient cohort (Fig. 2 and Table 4). In addition, this multiple biomarker model added a statistically significant increase in the predictive probability for clinical outcomes in patients with HF (AUC = 0.823, p = 0.0005) when it was tested on top of the BIOSTAT risk prediction model or as compared to the BIOSTAT risk prediction model alone (AUC = 0.643), respectively (Fig. 2 and Table 4). The increase in the AUC value between the BIOSTAT risk prediction model and the composite model of the BIOSTAT risk prediction model with the multiple peptide model was statistically significant.

Whilst some of these peptides could be derived from just one protein, it is likely that these fourteen peptides belong to several proteins. Identification of the peptides could provide more information about the pathogenesis in patients with HF in order to guide therapy. The multiple biomarker model of fourteen peptides may be useful if it could be applied for clinical practice. The prediction of clinical outcomes in patients with HF would be significantly improved using this multiple peptide biomarker model. Furthermore, the findings in this study demonstrated that there is a lot of predictive information in the proteomics which are not represented by the clinical factors and well-known biomarkers in the BIOSTAT risk prediction model. Therefore, proteomics mechanisms may improve our insight into the pathophysiological processes in HF that opens new perspectives for translational research in HF.

**Fig. 3:**
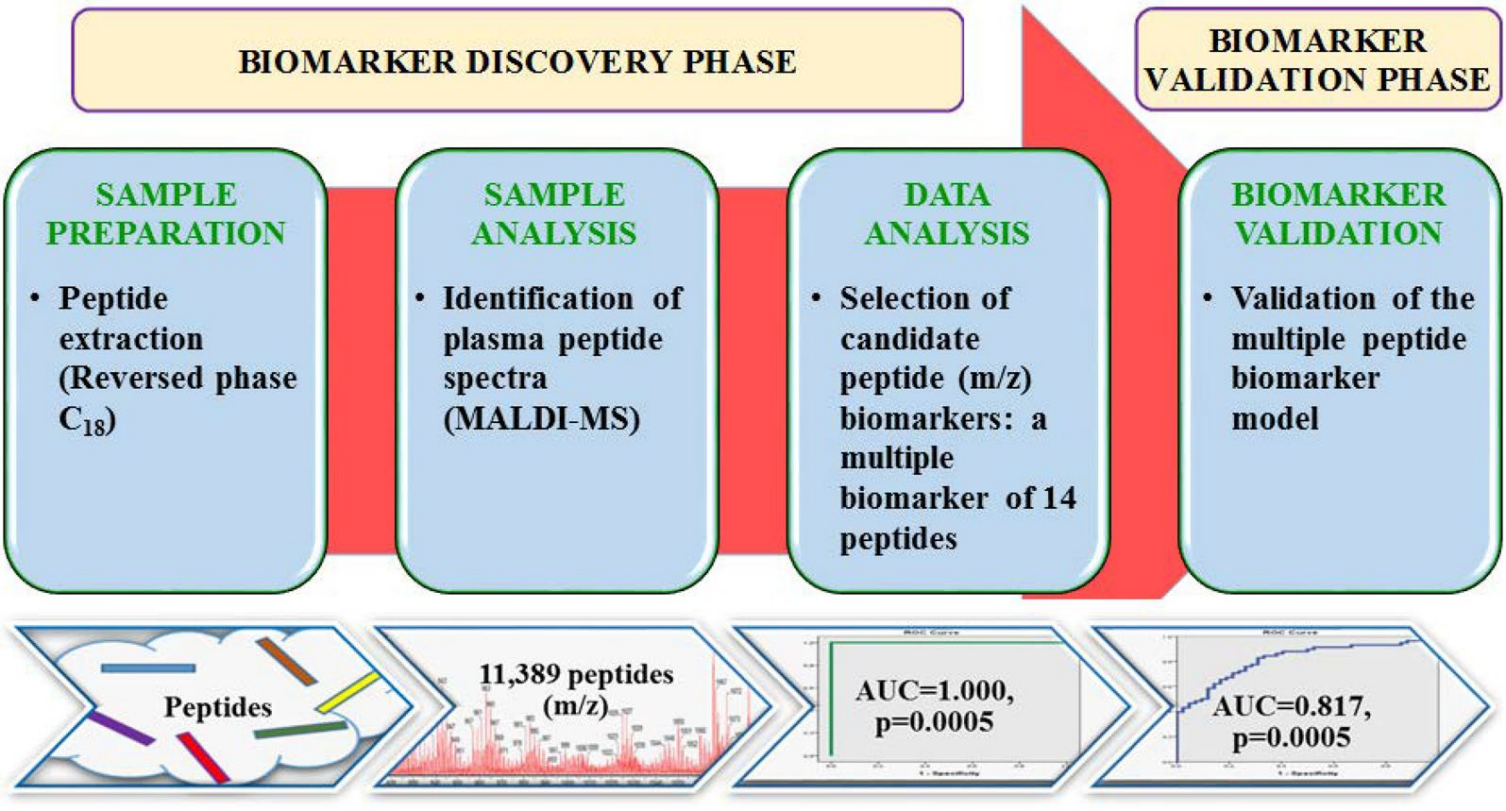
(Central illustration): Workflow of the biomarker discovery and validation phase for prediction of clinical outcomes in patients with heart failure using MALDI-MS

This is the first study using MALDI-MS profiling in order to predict clinical outcomes of patients with HF. The results obtained in this study demonstrated that MALDI-MS combined with C_18_ SPE technique is a good approach for discovery of potential biomarkers in plasma of patients with HF (Fig. 3: Central Illustration). This method also has the potential to provide insight into the pathophysiological processes in HF. MALDI is already established in some microbiology sections of clinical laboratories and consequently the expertise is already present to incorporate this kind of testing in the future [20, 21]. In addition, identification of clinical outcomes in HF that allow measurement of the disease on a peptide level. Therefore, this may result in their use in prognostication and selection of appropriate treatment in order to tailor therapeutics in HF [22].

A limitation of this study is that the mass spectrometer in our laboratory for MALDI technique only provides the m/z peptide peaks and their intensities to generate a profile for prediction of clinical outcomes in patients with HF, rather than identifying the underlying peptides or proteins. Another limitation of this study is that BIOSTAT-CHF project was exclusively Caucasian due to the study design. Therefore, the results of this study may only apply to patients of Caucasian ethnicity.

## Conclusions

In conclusion, to the best of our knowledge, this is the first study which discovered potential peptide biomarkers and a multiple peptide biomarker model for predicting clinical outcomes in patients with HF by using MALDI-MS combined with C_18_ SPE. The multiple peptide model in this study provided significant additional predictive information to the existing BIOSTAT risk prediction model. Further identification of these peptides may have important therapeutic implications for patients with HF in order to improve poor outcomes.

## Additional files

**Additional file 1: Table S1.** AUC values of 53 peptides (m/z) and the multiple biomarker model of fourteen peptides for prediction of clinical outcomes in the biomarker discovery HF patient cohort.

**Additional file 2: Figure S1.** Representative mass spectra of fourteen peptides (m/z) in the multiple biomarker model for prediction of clinical outcomes in patients with HF. There are m/z 2646.44, 2729.47, 3113.71, 5636.08, 5855.33, 5953.32, 6314.83, 6465.03, 6515.90, 7061.32, 7358.59, 7492.90, 7582.00 and 7929.78.

**Additional file 3: Figure S2.** Scatter 3D plot of fourteen peptides for predicting clinical outcomes in the biomarker discovery HF patient cohort. Each data sphere in the 3D plot corresponds to a patient with X-axis for treatment response, peptide (m/z) peak for the Y-axis, and Z-axis for the patient samples. This plot shows a very good separation between the HF patients who responded to treatment (green sphere) and HF hospitalisation or death (blue sphere).

**Additional file 4**: **Figure S3.** Predictive probability of time-to-event in patients with HF using the BIOSTAT prediction model, the prediction model of fourteen peptides and the combination model of prediction model of fourteen peptides and the BIOSTAT risk prediction.

## Abbreviations

AUC: area under the receiver operating characteristic curve
CI: confidence interval
EWP: extra wide pore
HF: heart failure
MALDI: matrix assisted laser desorption ionisation
MS: mass spectrometry
m/z: mass-to-charge ratio
ROC: receiver operating characteristic
SPE: solid phase extraction
