# Supplementary material for: Using matrix assisted laser desorption ionisation mass spectrometry (MALDI-MS) profiling in order to predict clinical outcomes of patients with heart failure
Source: Clin Proteomics. 2018 Nov 2;15:35. doi: 10.1186/s12014-018-9213-1 (PMC6214161; doi:10.1186/s12014-018-9213-1)
Supplement: Supplementary file 1 — Additional file 1: Table S1. AUC values of 53 peptides (m/z) and the multiple biomarker model of fourteen peptides for prediction of clinical outcomes in the biomarker discovery HF patient cohort. [file 12014_2018_9213_MOESM1_ESM.docx]

**Additional file 1: AUC values of 53 peptides (m/z) and the multiple biomarker model of fourteen peptides for prediction of clinical outcomes in the biomarker discovery HF patient cohort.**

| m/z | AUC | Standard error | p value | Asymptotic 95%  Confidence Interval | |
| --- | --- | --- | --- | --- | --- |
|  |  |  |  | **Lower Bound** | **Upper Bound** |
| 1724.22 | 0.623 | 0.056 | 0.034 | 0.267 | 0.487 |
| 2279.24 | 0.628 | 0.056 | 0.028 | 0.262 | 0.483 |
| 2290.24 | 0.617 | 0.056 | 0.043 | 0.273 | 0.493 |
| 2300.24 | 0.627 | 0.056 | 0.029 | 0.263 | 0.484 |
| 2410.29 | 0.627 | 0.057 | 0.028 | 0.261 | 0.485 |
| 2472.34 | 0.627 | 0.056 | 0.028 | 0.263 | 0.483 |
| *2646.44* | *0.636* | *0.056* | *0.019* | *0.527* | *0.745* |
| 2691.47 | 0.655 | 0.055 | 0.007 | 0.237 | 0.452 |
| *2729.47* | *0.621* | *0.057* | *0.037* | *0.267* | *0.490* |
| 2868.59 | 0.638 | 0.055 | 0.018 | 0.529 | 0.746 |
| *3113.71* | *0.618* | *0.056* | *0.042* | *0.508* | *0.728* |
| *5636.08* | *0.618* | *0.056* | *0.041* | *0.509* | *0.728* |
| 5660.99 | 0.614 | 0.056 | 0.049 | 0.504 | 0.725 |
| *5855.33* | *0.626* | *0.056* | *0.030* | *0.263* | *0.485* |
| *5953.32* | *0.651* | *0.056* | *0.009* | *0.542* | *0.760* |
| 6165.30 | 0.622 | 0.056 | 0.036 | 0.512 | 0.732 |
| 6279.13 | 0.632 | 0.056 | 0.023 | 0.522 | 0.741 |
| 6283.58 | 0.642 | 0.055 | 0.014 | 0.534 | 0.750 |
| *6314.83* | *0.625* | *0.056* | *0.031* | *0.515* | *0.735* |
| 6446.94 | 0.617 | 0.056 | 0.044 | 0.506 | 0.728 |
| 6460.55 | 0.628 | 0.056 | 0.027 | 0.519 | 0.737 |
| *6465.03* | *0.665* | *0.054* | *0.004* | *0.559* | *0.771* |
| *6515.90* | *0.688* | *0.054* | *0.001* | *0.207* | *0.417* |
| 6551.62 | 0.618 | 0.056 | 0.042 | 0.508 | 0.728 |
| 6576.58 | 0.666 | 0.054 | 0.004 | 0.561 | 0.772 |
| 6576.99 | 0.648 | 0.056 | 0.011 | 0.539 | 0.757 |
| 6601.97 | 0.616 | 0.056 | 0.046 | 0.506 | 0.726 |
| 6609.77 | 0.615 | 0.056 | 0.047 | 0.505 | 0.725 |
| 6722.04 | 0.650 | 0.055 | 0.010 | 0.542 | 0.759 |
| 6764.13 | 0.629 | 0.056 | 0.026 | 0.519 | 0.738 |
| 6918.14 | 0.624 | 0.057 | 0.033 | 0.513 | 0.735 |
| *7061.32* | *0.617* | *0.056* | *0.044* | *0.507* | *0.727* |
| 7100.13 | 0.626 | 0.056 | 0.030 | 0.517 | 0.735 |
| 7118.44 | 0.617 | 0.056 | 0.043 | 0.507 | 0.728 |
| 7121.74 | 0.611 | 0.057 | 0.056**^a^** | 0.500 | 0.722 |
| 7158.59 | 0.612 | 0.056 | 0.054**^a^** | 0.501 | 0.722 |
| 7185.63 | 0.625 | 0.057 | 0.031 | 0.514 | 0.736 |
| 7213.01 | 0.625 | 0.056 | 0.032 | 0.515 | 0.734 |
| *7358.59* | *0.644* | *0.055* | *0.013* | *0.536* | *0.753* |
| 7409.39 | 0.669 | 0.054 | 0.004 | 0.224 | 0.438 |
| 7463.58 | 0.642 | 0.056 | 0.015 | 0.531 | 0.752 |
| 7479.14 | 0.668 | 0.055 | 0.004 | 0.224 | 0.440 |
| *7492.90* | *0.627* | *0.056* | *0.028* | *0.263* | *0.483* |
| 7526.71 | 0.608 | 0.057 | 0.062**^a^** | 0.497 | 0.720 |
| 7572.41 | 0.612 | 0.057 | 0.053**^a^** | 0.502 | 0.723 |
| *7582.00* | *0.631* | *0.056* | *0.024* | *0.522* | *0.740* |
| 7600.74 | 0.635 | 0.056 | 0.020 | 0.527 | 0.744 |
| 7634.93 | 0.640 | 0.055 | 0.016 | 0.531 | 0.748 |
| 7649.22 | 0.616 | 0.056 | 0.046 | 0.505 | 0.726 |
| 7889.48 | 0.615 | 0.057 | 0.047 | 0.504 | 0.727 |
| 7914.92 | 0.650 | 0.055 | 0.010 | 0.542 | 0.758 |
| 7928.13 | 0.645 | 0.055 | 0.013 | 0.247 | 0.464 |
| *7929.78* | *0.616* | *0.056* | *0.045* | *0.506* | *0.727* |
| *Combined model*  *of 14 peptides* | 1.000 | 0.000 | 0.0005 | 1.000 | 1.000 |

***Peptides (m/z) in italic are peptides in the multiple biomarker model of fourteen peptides for prediction of clinical outcomes in the biomarker discovery HF patient cohort.***

***^a^ Peptides have AUC values with no significant p values.***
