# Supplementary material for: Using matrix assisted laser desorption ionisation mass spectrometry (MALDI-MS) profiling in order to predict clinical outcomes of patients with heart failure
Source: Clin Proteomics. 2018 Nov 2;15:35. doi: 10.1186/s12014-018-9213-1 (PMC6214161; doi:10.1186/s12014-018-9213-1)
Supplement: Supplementary file 2 — Additional file 2: Figure S1. Representative mass spectra of fourteen peptides (m/z) in the multiple biomarker model for prediction of clinical outcomes in patients with HF. There are m/z 2646.44, 2729.47, 3113.71, 5636.08, 5855.33, 5953.32, 6314.83, 6465.03, 6515.90, 7061.32, 7358.59, 7492.90, 7582.00 and 7929.78. [file 12014_2018_9213_MOESM2_ESM.docx]

**Additional file 2: Representative mass spectra of fourteen peptides (m/z) in the multiple biomarker model for prediction of clinical outcomes in patients with HF.** *There are m/z 2646.44, 2729.47, 3113.71, 5636.08, 5855.33, 5953.32, 6314.83, 6465.03, 6515.90, 7061.32, 7358.59, 7492.90, 7582.00 and 7929.78.*
