# Supplementary material for: Using matrix assisted laser desorption ionisation mass spectrometry (MALDI-MS) profiling in order to predict clinical outcomes of patients with heart failure
Source: Clin Proteomics. 2018 Nov 2;15:35. doi: 10.1186/s12014-018-9213-1 (PMC6214161; doi:10.1186/s12014-018-9213-1)

**Additional file 3: Scatter 3D plot of fourteen peptides for predicting clinical outcomes in the biomarker discovery HF patient cohort.** *Each data sphere in the 3D plot corresponds to a patient with X-axis for treatment response, peptide (m/z) peak for the Y-axis, and Z-axis for the patient samples. This plot shows a very good separation between the HF patients who responded to treatment (green sphere) and HF hospitalisation or death (blue sphere).*


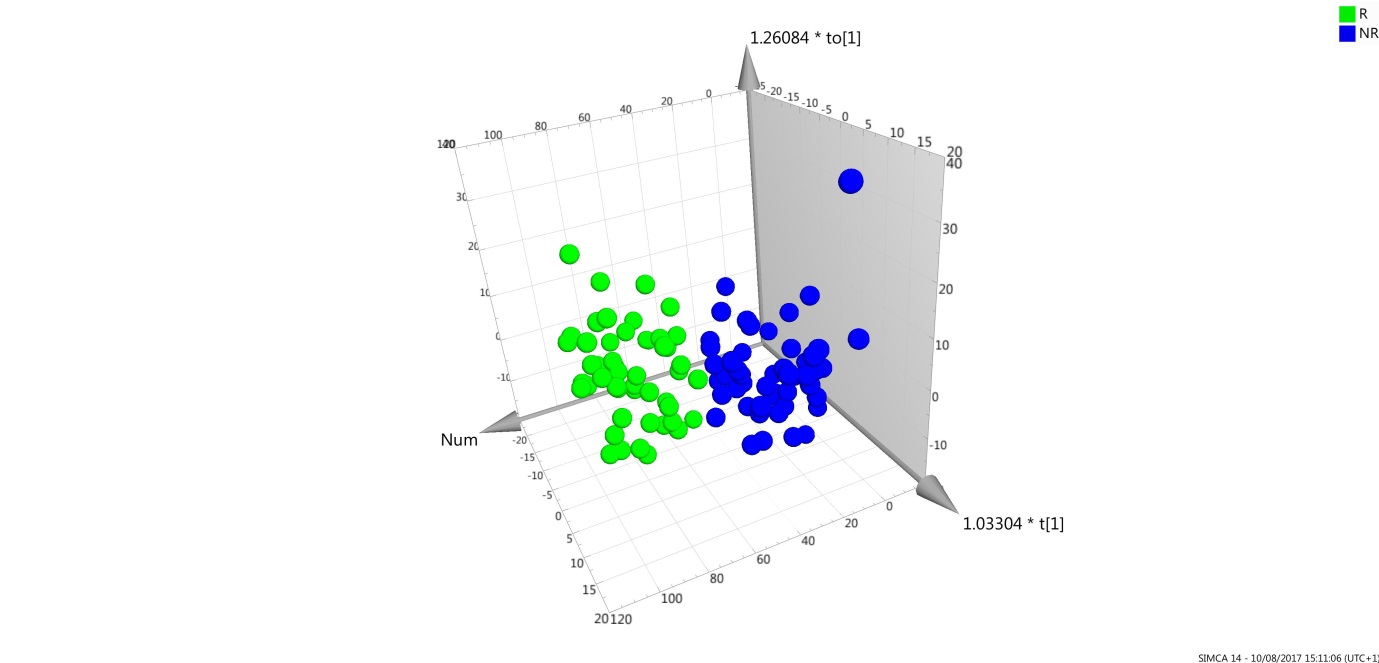

Supplement: Supplementary file 3 — Additional file 3: Figure S2. Scatter 3D plot of fourteen peptides for predicting clinical outcomes in the biomarker discovery HF patient cohort. Each data sphere in the 3D plot corresponds to a patient with X-axis for treatment response, peptide (m/z) peak for the Y-axis, and Z-axis for the patient samples. This plot shows a very good separation between the HF patients who responded to treatment (green sphere) and HF hospitalisation or death (blue sphere). [file 12014_2018_9213_MOESM3_ESM.docx]
