# Supplementary material for: Using matrix assisted laser desorption ionisation mass spectrometry (MALDI-MS) profiling in order to predict clinical outcomes of patients with heart failure
Source: Clin Proteomics. 2018 Nov 2;15:35. doi: 10.1186/s12014-018-9213-1 (PMC6214161; doi:10.1186/s12014-018-9213-1)
Supplement: Supplementary file 4 — Additional file 4: Figure S3. Predictive probability of time-to-event in patients with HF using the BIOSTAT prediction model, the prediction model of fourteen peptides and the combination model of prediction model of fourteen peptides and the BIOSTAT risk prediction. [file 12014_2018_9213_MOESM4_ESM.docx]

**Additional file 4: Predictive probability of time-to-event in patients with HF using the BIOSTAT prediction model, the prediction model of fourteen peptides and the combination model of prediction model of fourteen peptides and the BIOSTAT risk prediction.**

| 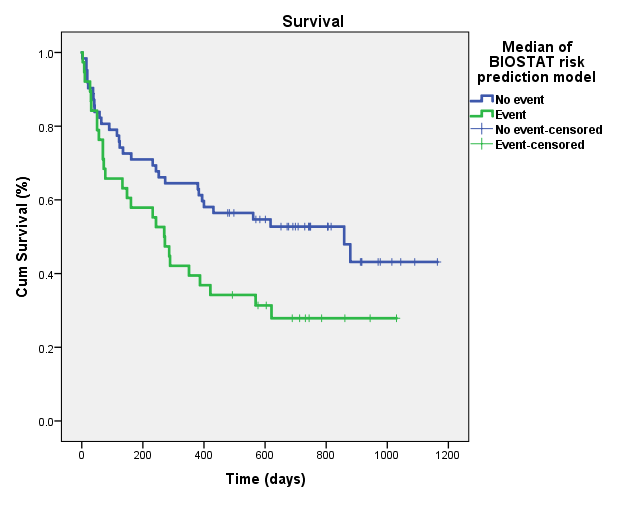 | **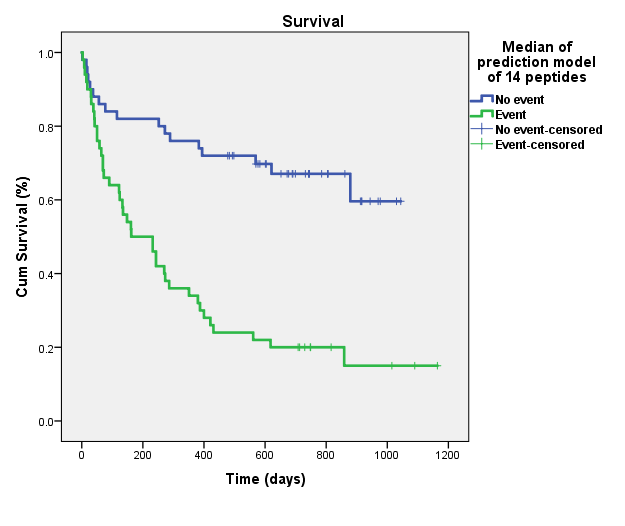** | 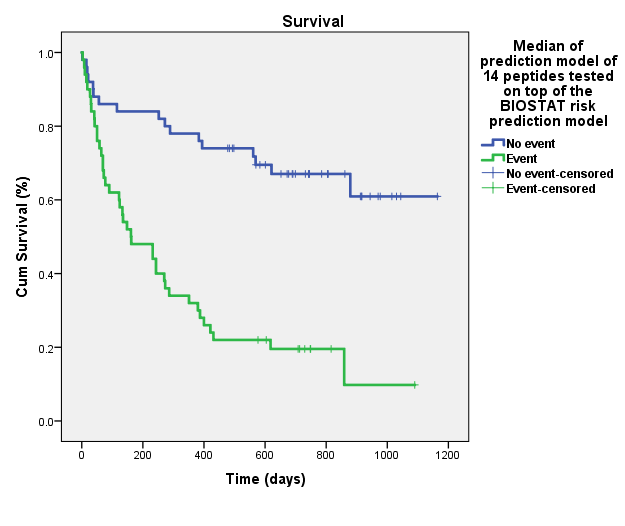 |
| --- | --- | --- |
